# Ocular Hygiene in Our Public Schools1Read before the Medical Society of the County of Erie, January 10, 1899.

**Published:** 1899-03

**Authors:** Benjamin F. Rogers

**Affiliations:** Buffalo, N. Y., Senior assistant-surgeon to the Charity Eye, Ear and Throat Hospital for Erie County, Fellow of Buffalo Academy of Medicine, Ophthalmological Society, etc.


### BUFFALO MEDICAL JOURNAL.

VOL. XXXVIII. MARCH, 1899. No. 8.

### Original Communications.

### OCULAR HYGIENE IN OUR PUBLIC SCHOOLS.[1]

BY BENJAMIN F. ROGERS, M. D., Buffalo, N. Y.,

Senior assistant-surgeon to the Charity Eye, Ear and Throat Hospital for Erie County, Fellow of Buffalo Academy of Medicine, Ophthalmological Society, etc.

IN THE statistics of our United States Educational report of the years 1896-'97, we find that there were in the various schools and colleges nearly 17,000,000 of pupils. Today we have in the City of Buffalo more than 72,000 pupils attending school—41,986 of this number in the public schools. About 23 per cent. of all school children have errors of refraction or other ocular troubles. A large percentage suffer from various forms of headache, vertigo and drowsiness—some, it may be observed, are very nervous, some have chorea, some one trouble and some another, nearly all, however, traceable to ocular defects.

Dr. Risley says "that great care should be exercised to avoid physical degeneracy during the years of school life, which are also the years of physiological growth. Observation has shown that a considerable percentage of those who enter upon the educational process in good health soon manifest impaired general vigor and develop eye troubles." It is plainly important, then, that the work in our schools should be so regulated that it shall rest as lightly as possible upon the young pupils. It is not the intention in presenting this paper to consider the subject of general hygiene, or to discuss ocular hygiene, further than to speak of those factors which have a more or less direct bearing upon the vision of school children. I therefore ask your attention for a few moments only to the nature and causes of impairment of vision during the years in the school-

1. Read before the Medical Society of the County of Erie, January 10, 1899.

room. It has been recognised for years that the requirements of school-life result in injury to the eyes of many children. In a number of cities some attention has been paid to the correction of errors of refraction of school children, yet we have not a well-defined and systematically applied plan of preventive measures existing in our schools. The correction of errors of refraction by the correct fitting of glasses to the eyes of such pupils as have trouble already existing should be one of the first measures to receive public attention. But you will agree with me that the ounce of prevention is to be applied by careful attention to the conditions surrounding the smaller pupils in the school-room. Here, in a multitude of cases, may be prevented the development of errors calling for glasses or complete debarment from study. From school reports of different cities I cannot find that a very great amount of work has been done in the city schools, except in a general way. In Germany the question has been studied in its most scientific bearings and the practical results applied in a systematic manner.

Dr. D. F. Lincalus says: "There is no hygienic point where the teacher can render more distinct service than in relation to the eyes of his pupils." The teacher realises that the eye is the *greatest* factor in education; not only in school but outside as well. Teachers have noticed that in many cases when efforts to draw the attention of pupils to their lessons have been fruitless, a defect in sight has been found, and this remedied, these scholars have kept pace with others."

Dr. Lee, before the American Academy of Medicine, in Philadelphia, in 1848, said: "Eyes originally sound are ruined in school by methods and conditions that are entirely avoidable."

Dr. Cohn, professor of ophthalmology in the University of Breslau, in 1865, examined the eyes of 10,060 school children. In his work on the Hygiene of the Eye in Schools, he says: "When I had begun my examination of the eyes of the Breslau school children, the question pressed itself upon me, how far may the old desks in our schools be answerable for the origin and development of school-sight?" To obtain an answer to this question he first of all measured the height of this vast number. He then measured the desks with reference to desk height (back and front), desk width, difference and distance between desk and form, between form and foot-board. He thus found that these desks were *opposed* to every reasonable hygienic requirement. Pupils three feet six inches and five feet two inches in height sat at the same desk. Apart from this fundamental error he found the pupils, even when the desk was suited to their height, were forced to stoop forward and bring the eyes very close to the writing. That is just the way eye trouble (especially myopia) can be produced and increased.

"Ocular hygiene," says Morton, "largely resolves itself into a matter of eliminating all factors, about the pupil and his work, which cause strain, fatigue and congestion." These are the factors which excite trouble in eyes predisposed and develop trouble in normal eyes.

Some of our old school-rooms in this city are nothing more nor less than hot-beds for eye troubles—turnstiles for the emetropic ball to the hypermetropic ball, that over into the myopic ball. The principal abnormal conditions which we meet in the eyes of school children are the short-eye or hypermetropia, hypermetropia with astigmatism, and the long-eye or myopia—children with hypermetropia or hyperopic astigmatism, or both, I fit with glasses at once and have them come to my office at least once a month during the school session. The pupils with myopia, which is an acquired condition almost without exception, should be debarred from all reading and study for a time, suitable glasses, not too strong, should be adjusted and the oculist have full care and control of the eyes for at least a year or even during school-life. Myopia is called a disease, which cannot be cured and often cannot be arrested. Progressive myopia is in every case ominous of evil for the future, for if it continues the eye soon becomes less and less equal to its work. Many statistics could be brought forward to prove the last two or three statements, were it my intention to enter upon the critical study of myopia. The most dangerous to the eye are the four higher grades in our grammar schools, leading up to the high schools. It is there that myopia commences and increases. There you will find myops as you ascend the grades—32½ per cent. of the pupils wearing glasses in the high schools are myops.

Cohn, in his study of seating in its bearing upon myopia in the public schools, states: "That in every school the number of myopic pupils increased from grade to grade. He attributed this to improperly adjusted desks. The adjusting of each desk to the pupil occupying it and not the pupil to the desk is the important point. Extensive statistics of school examinations could be presented, but a few will suffice at this time. In Cohn's statistics the following progression in the percentage of myopia was shown in the various schools examined by him: Five village schools, 1.4 per cent.; twenty elementary schools, 6.7 per cent.; two higher girls' schools, 7.7 per cent.; two intermediate schools, 10.3 per cent.; two Realschulen, 19 per cent.; two gymnasiums, 26.2 per cent. Among university students he found that the percentage of myopic eyes had advanced to 59.5 per cent. This shows conclusively that the number of short-sighted pupils increases from the lowest to the highest schools. My statistics of all the public schools of this city are not complete. However, nineteen of the largest schools in different parts of the city, with nearly 20,000 pupils, furnish us ample material for our percentages for all. We find 4 per cent. or 1,700 wearing glasses—19½ per cent. or 7,787 having some form of eye trouble, who should be examined at once, and if needing glasses, be advised of the fact as soon as possible. In school No. 3, or the Perry Street School, with a registration of 700, we find only three wearing glasses, while the principal, Professor Ryan, says: "We have many children whose eye-sight is poor, but who are unable to procure glasses."

School No. 31, or Emslie Street School, the largest public school in the city, with a registration of 2,650, sixty pupils wear glasses.

School No. 19, or West Avenue School, registration 1,089, we find eighteen in the primary department; sixteen in the intermediate department and seventeen in advanced department, A total of fifty-one wearing glasses.

School No. 45, or Auburn Avenue School. First grade omitted. Second grade, with registration 112, not any wear glasses; 21 need the examination on account of eye trouble. Third grade, registration 160, five wear glasses; 50 need the examination. Fourth grade, registration 114, 6 wear glasses; 36 need examination. Fifth grade, registration 122, 6 wear glasses; 37 need examination. Sixth grade, registration 111, 6 wear glasses; 15 need examination. Seventh grade, registration 93, 8 wear glasses, 37 need examination. Eighth grade, registration 65, 5 wear glasses; 38 need examination. Ninth grade, registration 70, 6 wear glasses; 22 need examination. Total registration, 847—40 wear glasses; eye trouble, 256.

Masten Park High School, registration 1,181—480 boys, 701 girls; 49 boys and 114 girls wear glasses—58 of this number wear myopic glasses or more than 35 per cent. Central High School, registration 863—105 pupils wear glasses; 29 of this number wear myopic glasses, or more than 27 per cent. 32½ per cent. is the average percentage wearing myopic glasses. 15 per cent., or almost one-half of the average percentage ($32\frac{1}{2}$) wear a compound myopic glass. 13 per cent. of the total number in the high schools wear glasses. Lack of knowledge on the subject of ocular hygiene may be partly responsible for the condition prevailing in our schools. A few of many cases which have come under my observation afford illustration :

A lad, aged 12, from one of the public schools, came to my office accompanied by his mother. The mother stated that her boy would not go to school; that he complained of his eyes, that he could not see, and the like. She said that she had bought him a pair of good glasses on Elk street for fifty cents. The boy still insisted that he could not see to read and the mother still declared that he was trying to deceive her, giving as her reason for this belief that she could see all right herself and could not understand why the boy could not see. Upon examination I found his vision to be $\frac{20}{20}$; with the proper glass correcting his compound hyperopic astigmatism, his vision became $\frac{20}{20}$.

A young man, aged 17 years, commenced school at the age of 7 with normal vision. At the age of 13 years had to stop school on account of progressive myopia. His vision now is less than $\frac{20}{200}$. What did the mother of this boy do? She related the case to a traveling jeweler, who sold her a strong far-sighted glass for the boy, and she tried for two years to make him wear them.

A large percentage of children are entered without question as to the state of their vision, due in measure to want of time on the part of the teachers. Educators accept the important trust committed to them, not knowing whether the vision of the pupil is such as to endure his work in the school-room. Many parents do not know that the vision of their children is defective or may soon become so, and in consequence their health be endangered and their educational progress retarded. With these facts before us, what can and should be done in the way of ocular hygiene in the public schools; yes, all schools? The board of health, the superintendent of education, the teachers, the oculists, all physicians, the press—in fact, the general public should take up this subject and avoid as far as possible injuring the eyes and health of the pupils while carrying on their instruction in our schools. Popular education is demanded. Our first duty is to place specific guardianship over the pupils who enter upon their educational process with defective eyes; a more elastic curriculum should be provided for such pupils. A systematic method of inspection should be adopted and commenced at once in our schools, which will be effective in detecting the factors which are detrimental to the eyes. The eyes of each pupil should be examined with the utmost care that the state of refraction may be ascertained and a record kept which, when needed for reference, would be strictly accurate in all statements of fact. In order to keep such a record a blank form should be used for noting all principal points of each case, viz., name, age, sex, color of eyes, whether eyes are comfortable or weak when used, the presence of any form of inflammation, corneal opacity or strasbismus; the sharpness of vision of each eye, as shown by Snellen's or some other standard test-type, the presence or absence of astigmatism and the range of accommodation. The eyes of all children should be tested before admission as pupils to the school. If the vision should prove to be much below the normal, the parents should be advised as to this condition and what should be done. Admission should not be granted until the pupil has sought professional counsel and presents a certificate to that effect. The same rule should apply to children with inflamed eyes, who should not be allowed in the school until a physician's certificate of the noninfectious nature of the disease has been presented. The common wash-basin, towel and comb should be abandoned entirely.

Can the teacher make this nonprofessional test? Yes. The teacher should be able to determine whether the pupil has normal vision or not. A chart of standard test letters, an astigmatic chart and a card of diamond type for the near-point, with simple instructions as to their use should be placed in each school-room. Can all parents of children with defective vision pay for an examination and for glasses? We have a great many pupils in our schools whose parents cannot pay for the examination required at the oculists' office, and who will not for various reasons avail themselves of relief at free institutions for such cases. Quite a number, however, of our school children suffering from headache or eye-strain do come to these institutions, and we know that the oculists who work there day after day, year in and year out, are more than willing to examine all worthy cases. At the Charity Eye and Ear and Throat Hospital, scarcely a day passes without several presenting themselves for examination for glasses. Some get their glasses at once, yet I have known others to delay from three to six months, and why? Because their parents could not pay for glasses. It is my judgment that, if there is money in the public crib for a speedway, surely some funds ought to be available to provide glasses for the child with defective vision, who is struggling through the precious hours of youth trying to fit himself for the responsibilities of life.

Then again, in our schools we find a certain number called "dull" or "backward," and why is it? Not all of these are drones, by any means. In one school there is a class of twenty-four pupils in one room selected from the different grades, sixteen of the twenty-four, or 66⅔ per cent., have headache most of the time. Ten, or more than 41 per cent., cannot read by artificial light. Is it not reasonable to believe that if this class of pupils could be examined for errors of refraction, we should find a large percentage needing glasses? I repeat, that school children needing glasses should be examined at once and the correct glass adjusted.

The application of ocular hygiene in the school room and to the pupils, in accordance with modern scientific methods, would go far to arrest the acquisition of near-sight and its attending pathological conditions. The factors demanding attention in the school rooms, which I will touch on very briefly, are: Light—quantity and quality—tinted walls, desks, artificial light, writing on black boards, excessive work, and length of school year.

*Light.*—This commences, of course, with location of school lot, its surroundings, the number and location of windows. Quantity and quality of light are modified by the color of walls and shades to the windows. Shades should be hung on the adjustable shade fixtures.

*Tints.*—Blue, gray or neutral tints are best for walls.

*Desks.*—Adjustable desks should be used and placed so that the light falling from the upper sash, when possible, will strike the desk over the pupil's left shoulder.

*Artificial Light.*—Artificial light is always a bad light for young eyes; school children with myopia or any form of eye-strain should not work or study by artificial light.

*Writing on Black Boards.*—The writing should be large and legible; if required to be read at fifteen feet, should be large enough to be read at thirty feet.

*Excessive Work.*—School hours should be carefully adjusted to the strength of the pupil. There should be frequent intervals during school hours for relaxation of the eyes.

*Length of School Year.*—There is no time gained for the pupil by school sessions the last half of the month of June and first half of September, the two most beautiful months of the year for out-door recreation. There is a wide opportunity for applying ocular hygiene in our schools. A great many unfavorable factors have been eliminated by way of architecture. If the graded system is to be continued, there should be more and larger rooms. In some of the rooms the aisles between desks are only eleven inches, and sixty pupils in a room intended for only forty. Our superintendent of public instruction has lessened and is lessening the number of objectionable factors, yet, reviewed from the standpoint of ocular hygiene, there is plenty to do. I am greatly indebted for courtesies extended me by our superintendent of public instruction and by many of the principals and teachers of our schools, who have aided me materially. I trust I have demonstrated, though in a very imperfect way, how intimately connected the factors this paper has mentioned are with ocular troubles in our schools.

221 FRANKLIN STREET.

